# Color-Change Detection Activity in the Primate Superior Colliculus

**DOI:** 10.1523/ENEURO.0046-17.2017

**Published:** 2017-04-12

**Authors:** James P. Herman, Richard J. Krauzlis

**Affiliations:** Laboratory of Sensorimotor Research, National Eye Institute, Bethesda, MD 20982-4435

**Keywords:** change detection, color, spatial attention, superior colliculus

## Abstract

The primate superior colliculus (SC) is a midbrain structure that participates in the control of spatial attention. Previous studies examining the role of the SC in attention have mostly used luminance-based visual features (e.g., motion, contrast) as the stimuli and saccadic eye movements as the behavioral response, both of which are known to modulate the activity of SC neurons. To explore the limits of the SC’s involvement in the control of spatial attention, we recorded SC neuronal activity during a task using color, a visual feature dimension not traditionally associated with the SC, and required monkeys to detect threshold-level changes in the saturation of a cued stimulus by releasing a joystick during maintained fixation. Using this color-based spatial attention task, we found substantial cue-related modulation in all categories of visually responsive neurons in the intermediate layers of the SC. Notably, near-threshold changes in color saturation, both increases and decreases, evoked phasic bursts of activity with magnitudes as large as those evoked by stimulus onset. This change-detection activity had two distinctive features: activity for hits was larger than for misses, and the timing of change-detection activity accounted for 67% of joystick release latency, even though it preceded the release by at least 200 ms. We conclude that during attention tasks, SC activity denotes the behavioral relevance of the stimulus regardless of feature dimension and that phasic event-related SC activity is suitable to guide the selection of manual responses as well as saccadic eye movements.

## Significance Statement

Control of spatial attention by primate superior colliculus (SC) is traditionally viewed as supporting orienting or targeting behaviors, and has been tested with a limited set of visual features. Additionally, despite SC’s characterization as a novel event detector, analysis of SC neuronal activity has been limited to stimulus onset or delay periods. Here, we document change-detection activity in SC: large phasic bursts shortly after threshold-level isoluminant color saturation changes during a task requiring maintained fixation and manual joystick response. Change-detection activity is modulated by spatial cueing, but is also predictive of response choice and accounts for a substantial proportion of response latency. Activity in SC may thus guide the selection of motor responses to visual events regardless of effector or feature dimension.

## Introduction

There is ample evidence that the primate superior colliculus (SC) is important for the control of spatial attention. Intermediate-layer SC neurons exhibit cue-related modulation during attention tasks (Kustov and Robinson, 1996; Ignashchenkova et al., 2004). Microstimulation produces a spatially specific improvement in detection performance at retinotopic locations corresponding to the stimulation site (Cavanaugh and Wurtz, 2004; Müller et al., 2005). Conversely, reversible inactivation causes deficits in attention for stimuli inside the inactivation-affected area of the visual field, as well as difficulty in suppressing responses to distractor stimuli outside that area (Lovejoy and Krauzlis, 2010, Zénon and Krauzlis, 2012).

Several crucial aspects of the SC’s role in spatial attention are unclear. First, studies establishing a causal role for the SC in controlling spatial attention have all used motion (Cavanaugh and Wurtz, 2004; Müller et al., 2005; Lovejoy and Krauzlis, 2010; Zénon and Krauzlis, 2012). This is problematic because motion may have a privileged status in the SC. Several studies have identified motion-sensitive neurons in the SC, although direction selectivity is uncommon (Marrocco and Li, 1977; Moors and Vendrik, 1979; Davidson and Bender, 1991; Krauzlis, 2004). Because the SC is an evolutionarily conserved structure for orienting, the SC may be particularly sensitive to motion because of its salience in predatory-prey interactions.

Second, recording studies have not characterized SC neuronal activity during stimulus events like those used in causal attention experiments (Cavanaugh and Wurtz, 2004; Lovejoy and Krauzlis, 2010; Zénon and Krauzlis, 2012) but, instead, have focused on cue-related modulation. One of the ways that SC activity might contribute to the performance of attention tasks is through the detection of behaviorally relevant events. Indeed, there is evidence that the SC plays a functional role in event-detection in frogs (Lettvin et al., 1961; Gaillard, 1990) and rats (Comoli et al., 2003; Dommett et al., 2005). If similar event-detection activity were present in the primate SC, it could play a central role in the performance of attention tasks.

Third, in most attention studies, monkeys indicate their choice with saccades, introducing possible confounds between attention-related modulation and the effects of saccade planning in the SC. Previous studies have addressed this problem by focusing on epochs of the trial after attention has been cued, but before the choice stimulus has been presented, minimizing the contribution of neuronal activity related to the planning and execution of saccades (Ignashchenkova et al., 2004). However, this approach precludes analysis of possible attention-related effects during the stimulus event that guides the behavioral choice.

In the current study, we addressed these limitations by recording from SC neurons during the performance of an attention task using a novel color stimulus rather than motion, and had monkeys indicate their choice with their hands rather than their eyes. We used a dynamic color stimulus similar to that used in a study of attention in human subjects (Herman et al., 2015); monkeys were required to detect near-threshold changes in the color saturation of a peripheral stimulus and respond with a joystick while maintaining central fixation. Because the processing of color signals involves circuits very different from those involved in motion processing, in particular, detecting changes in color saturation is unlikely to be accomplished without cortical processing, a task with color stimuli provides a good test of whether SC’s role in attention extends to other visual features.

Our results demonstrate substantial attention-related modulation with color stimuli, as well as modest color preferences, both of which varied in magnitude during the trial. Most striking, however, was the phasic activity evoked by threshold-level changes in color saturation, which was as large as the activity evoked by stimulus onset. This activity was predictive of the monkey’s choice to release or hold the joystick, and accounted for a substantial proportion of joystick reaction time (jRT). Thus, beyond its specific roles in processing motion and saccades, the primate SC may signal behaviorally relevant events for the selection of actions in general.

## Materials and Methods

### General

Data were collected from two adult male rhesus monkeys (*Macaca mulatta*) weighing 9–12 kg. All experimental protocols were approved by the National Eye Institute Animal Care and Use Committee and all procedures were performed in accordance with the United States Public Health Service policy on the humane care and use of laboratory animals.

### Tasks

Monkeys were seated in primate chairs (Crist Instrument) with their heads fixed in a darkened booth. Primate chairs were modified so that a joystick extended toward the animal from the center of the front face of the chair. Gaze was measured with an Eyelink 1000 infrared video tracking system (SR Research), and experiments were orchestrated with a modified version of PLDAPS (Eastman and Huk, 2012).

**Figure 1.**
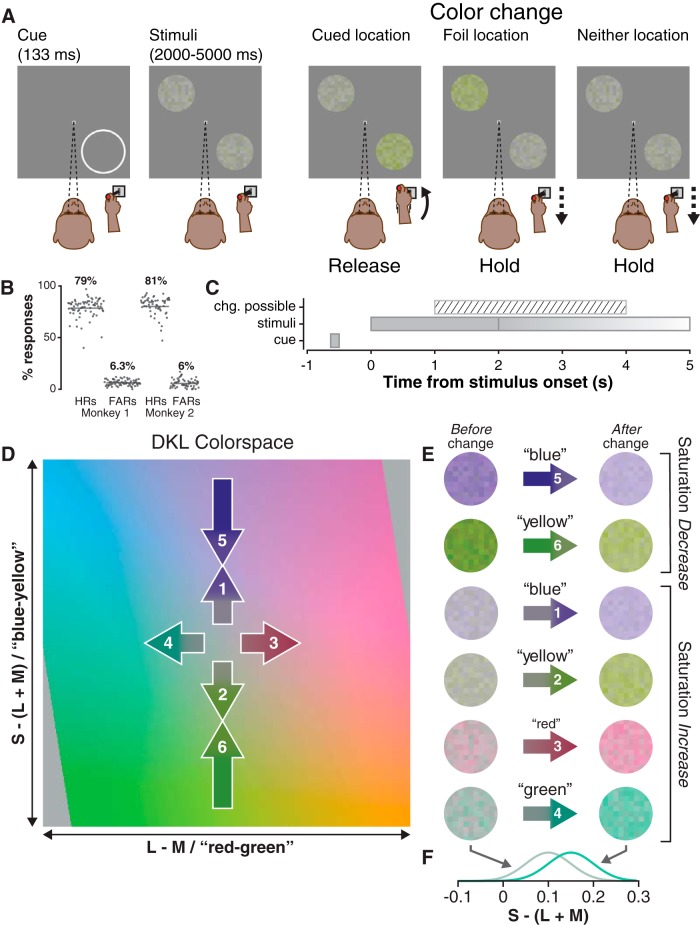
Attention task procedure, stimuli, and behavior. ***A***, Procedure: the monkey was required to hold down the joystick and maintain central fixation throughout the trial, releasing his hold only for cued stimulus changes. The cued stimulus patch was presented in the location previously occupied by the flashed cue-ring (the bottom right of the screen, in this example), and the foil was presented at an equally eccentric opposing location (top left). Only one stimulus changed per trial (see Materials and Methods). ***B***, Hit rates (HRs) and false-alarm rates (FARs) for monkey 1 and 2 in each session; percentages are across-session binomial parameter estimates and horizontal lines are 95% confidence intervals. ***C***, Trial timeline illustrates the time window in which a color change was possible relative to stimulus presentation; stimuli were presented for 1 s beyond the time of the color change. D, Stimulus colors were drawn from an isoluminant plane of the DKL color space. Axes were scaled to the [−1, +1] interval with limits representing the maximum possible contrast for the display (see Materials and Methods). Numbered arrows (not to scale) represent color changes in saturation increase (1, 2, 3, 4) and saturation decrease (5, 6) trials; saturation decreases were approximately two to four times larger than saturation increases and were arranged so that the mean saturation was the same following either an increase or a decrease. ***E***, Example stimulus frames before (left) and after (right) saturation increases (1, 2, 3, 4) and decreases (5, 6). Note that after a blue increase (1) or a blue decrease (5), the saturation was the same, as mentioned above; the same was true for yellow increase (2) and decrease (6). ***F***, Example saturation distributions for stimuli before and after a green saturation increase; before increase, Gaussian distribution has (mean, SD) of (0.1, 0.05) and (0.15, 0.05) after increase.

Attention task trials began when the monkey pressed down on the joystick, triggering the appearance of a 0.25° wide white fixation square (48 cd/m^2^) against a gray background (28.5 cd/m^2^), which the monkey was required to fixate (1° window) within 1 s of its appearance (Fig. 1*A*); fixation was required until reward delivery, and joystick press was required until a stimulus change or reward delivery. After 500 ms of fixation, a white cue-ring (48 cd/m^2^), was presented [8–11° eccentric depending on receptive field (RF) eccentricity] for 133 ms (inner radius 3.75°, outer radius 4°). Two stimulus patches (3.25° radius) were then presented 500 ms after cue offset (Fig. 1*C*); the “cued” patch was centered on the former location of the cue-ring, and the “foil” patch was presented at an equally eccentric opposing location: 180° away. After 68 trials with this configuration, the location of the cue-ring was switched to the opposing, 180° away location. A saturation change was possible 1–4 s after stimulus onset (uniform distribution), and stimuli were extinguished 1 s after the change for a total presentation time of 2–5 s. Because of the dynamic nature of the stimulus (see below), the change itself had a duration of 80 ms. If the cued stimulus changed, the monkey was required to release the joystick within a time window 175–750 ms after the change; if the foil or if neither stimulus changed, the monkey was required to maintain joystick press for 1000 ms after the change (or after an identically distributed “sham change time” if neither stimulus changed). If the monkey correctly released the joystick for a cued change or correctly maintained his hold for a foil/neither change, he was given a liquid reward 1000 ms after the stimulus change (or after the sham change), ending the trial. For monkey 1, only one of the two stimuli (cue or foil) could change in each trial, not both; we modified the task structure for monkey 2 to correct an inappropriate behavioral strategy that was not a problem with monkey 1. The results focus on the trial conditions that were identical in both monkeys.

In addition to the main attention task, a visually guided saccade task was used to map RFs and a memory-guided task was used to classify neurons. In these tasks, saccades were detected using a 25°/s threshold on an online estimate of gaze velocity. In visually guided saccade trials a 0.25° wide green fixation square (22 cd/m^2^) appeared in the center of the screen against a gray background (28.5 cd/m^2^). The monkey was required to maintain his gaze within a 1–1.5° window around this point until it was extinguished. A 0.25° wide white target square appeared after 500–750 ms, and the fixation was extinguished 1000–1500 ms later, indicating that the monkey should saccade to the target. The monkey was required to start his saccade 100–400 ms after target extinction, had to land within 2–5° of the target (adjusted online depending on target eccentricity), and had to maintain fixation on the target for 375–750 ms to obtain his liquid reward. In memory-guided saccade trials, the fixation square was instead red (23.2 cd/m^2^). After 1000–1500 ms, the target square was flashed for 100 ms, and the fixation remained on for a further 500–1000 ms before extinguishing. The monkey was required to saccade to the remembered location of the target 100–600 ms after fixation extinction and had to land within 3–6° of the target location to trigger the reappearance of the target stimulus before maintaining target fixation to obtain reward.

### Stimuli

Stimuli were displayed at 1920 × 1200 resolution (∼60 × 38°), 100-Hz frame-rate on a VIEWPixx display (VPixx Technologies) controlled by a mid-2010 Mac Pro (Apple) running MATLAB R2012b (The Mathworks) with the Psychophysics Toolbox extensions (Brainard, 1997; Pelli, 1997; Kleiner et al., 2007). Display luminances and chromaticities were measured with a Tektronix J18 LumaColor II Photometer with J1803 Luminance Head and J1810 Chromaticity Head (Tektronix).

Stimulus colors were defined on an isoluminant plane of the DKL color space (Derrington et al., 1984). Conversion between DKL and RGB was achieved by a method described previously (Herman et al., 2015). DKL space was defined using cone contrasts computed from monitor CIE xyY coordinates and the cone fundamentals of Smith and Pokorny (1975). The xyY coordinates for our display were: R (0.68, 0.32, 23.0), G (0.13, 0.71, 40.0), B (0.14, 0.08, 6.4). We scaled each axis of DKL space between −1 and +1, with the ends of this interval corresponding to the maximum possible color contrast on the display used (Hansen et al., 2008).

Our color stimuli were inspired by “random dot kinematogram” motion stimuli (Britten et al., 1992), in that they were dynamic, stochastic, and used fixed-lifetime elements. They consisted of circularly windowed 11 × 11 “checkerboards” of 18-pixel checks (Fig. 1*E*); windowing kept 109 unique checks. Each check’s color was drawn from a 1D Gaussian (SD = 0.05) on an isoluminant plane of DKL color space ([27.8 cd/m^2^, 28.2 cd/m^2^]; Fig. 1*D*), so that they varied in saturation but not hue; check luminance was then independently set to a value in the interval: [26.5 cd/m^2^, 29.5 cd/m^2^]. Thus, regardless of hue or saturation, stimuli were (on average) physically isoluminant with the background, but also had nonzero contrast due to check-to-check variation in luminance. Each check had a lifetime of eight frames (80 ms), during which its color and luminance were static. At the end of a check’s lifetime, new color and luminance values were drawn, and its frame counter was reset. In the first frame of stimulus presentation, check counters were initialized with random “age” values between 1 and 8, so on each subsequent frame only a subset (∼1/8) of checks was reborn. An important consequence of check lifetime is that saturation changes were not complete until all of the checks had been “reborn” eight frames (80 ms) after change onset.

Saturation changes were increases or decreases in the mean of the Gaussian distribution from which color values were drawn (Fig. 1*F*). In saturation increase trials the mean of the underlying Gaussian distribution was initially 0.1 from the origin (white point) along one of the two axes; at change-onset, the mean was shifted radially outward by 0.03–0.05 so the color of checks born after change-onset would be drawn from a Gaussian with a larger mean (Fig. 1*D–F*). The magnitude of the change in mean saturation was chosen to keep the monkeys performance at ∼80% (Fig. 1*B*); saturation decrease magnitudes had to be two to four times larger than increase magnitudes to maintain comparable performance. In sessions with both saturation increases and decreases, the pre-change mean saturation in decrease trials was chosen so that the post-change mean saturation would be identical for increase and decrease trials (Fig. 1*D*, numbered arrows). Saturation decreases were used in a minority of sessions (*n* = 22) and were presented in blocks of trials that alternated with saturation increases.

### Electrophysiological recordings

Sharp epoxy-coated tungsten electrodes (FHC) were introduced into the SC with a motorized microdrive (Narishige), and putative action potential waveforms were digitized and saved with a Plexon MAP system (Plexon). Once a neuron was stably isolated, the visual saccade task was used to map its RF: a 2D Gaussian was fit to the responses in real-time using an extension of PLDAPS running in MATLAB. Next, 15–30 trials of the memory-guided saccade task were used to characterize the neuron’s baseline, visual, delay, and saccadic activity with the saccade target placed at the center of the neuron’s RF (or movement field if it was not visually responsive). Next, attention task data were gathered, initially placing either the cued or the foil patch such that it overlapped with the RF center. As mentioned above, the location of the cue-ring was alternated every 68 trials so that cue and foil were presented in the neuron’s RF in alternate blocks. After the attention task, the memory guided saccade task was repeated to confirm the neuron’s identity. All spike data were sorted offline.

### Neuron type categorization

We used spike rates from four time windows in the memory guided saccade task to categorize neurons (McPeek and Keller, 2002): baseline (−75 to +25 ms from target onset), visual (+50 to +200 ms from target onset), delay (−150 to +50 ms from fixation offset), and saccade (−25 ms from saccade onset up to saccade offset). Saccade onset and offset times were calculated offline using combined velocity and acceleration criteria and verified by inspection. We then computed a one-way Kruskal-Wallis nonparametric ANOVA using spike rates in each window for each trial and used *post hoc* testing to determine the presence of significant visual, delay, or saccade activity compared with baseline. Neurons with significant visual, delay, and saccade activity were labeled “visual-movement prelude” (VMp) (*n* = 26), those with significant visual and delay activity were “visual-delay” (*n* = 9), significant delay and saccade activity were “movement-delay” (*n* = 8), significant visual and saccade were “visual-movement” (VM) (*n* = 45), significant visual only were “visual” (V) (*n* = 42), and significant saccade only were “movement” (*n* = 9).

### Data analysis

Analyses of covariance (ANCOVAs) were performed in R (R Core Team, 2016), all other analyses were performed in MATLAB. A summary of statistical tests can be found in Table 1. 

**Table 1.** Statistical tests

Test use	Test	Data structure	Power
Individual neuron color preferences (Fig. 2)	ROC area significance bootstrapping	Non-Gaussian	95% CI > 0.5
Population percent color preference (Figs. 2B, D, 3D)	χ2-proportion test	Proportion	*p* < 0.05
Individual neuron cue/foil preferences	ROC area significance bootstrapping	Non-Gaussian	95% CI > 0.5
Unit type category AMI comparison (Fig. 3B)	ANOVA	Gaussian	α = 0.05, *p* < 0.05
Population percent cue/foil preference (Fig. 3C)	χ2-proportion tests	Proportion	*p* < 0.05
Time course of cue and color preferences (Fig. 3C,D)	ANCOVA	Gaussian	α = 0.05, *p* < 0.01
Presence of significant change-detection activity in single neurons	Wilcoxon rank sum test	Non-Gaussian	*p* < 0.05
Comparison of onset-evoked and change-evoked peak firing rates	ANVOA	Gaussian	α = 0.05, *p* < 0.05
Individual neuron saturation increment vs. decrement preferences (Fig. 5)	ROC area significance bootstrapping	Non-Gaussian	95% CI > 0.5
Individual neuron hits vs. misses preferences (Fig. 6)	ROC area significance bootstrapping	Non-Gaussian	95% CI > 0.5
Unit type category detect-probability comparison (Fig. 6)	ANOVA	Gaussian	α = 0.05, *p* = 0.77
Unit type category percent hits preference comparison (Fig. 6)	χ2-proportion tests	Proportion	*p* > 0.05
Comparison of hinge model component contributions to variation in threshold time	ANOVA	Gaussian	α = 0.05, *p* < 0.01

Average firing rate traces were computed by convolving individual spike trains with a fast rising (τ = 1 ms), slow falling (τ = 20 ms) combination of exponentials resembling a postsynaptic potential (Thompson et al., 1996). In all figures and for all analyses, stimulus-onset-aligned data were truncated at change-onset.

**Figure 2.**
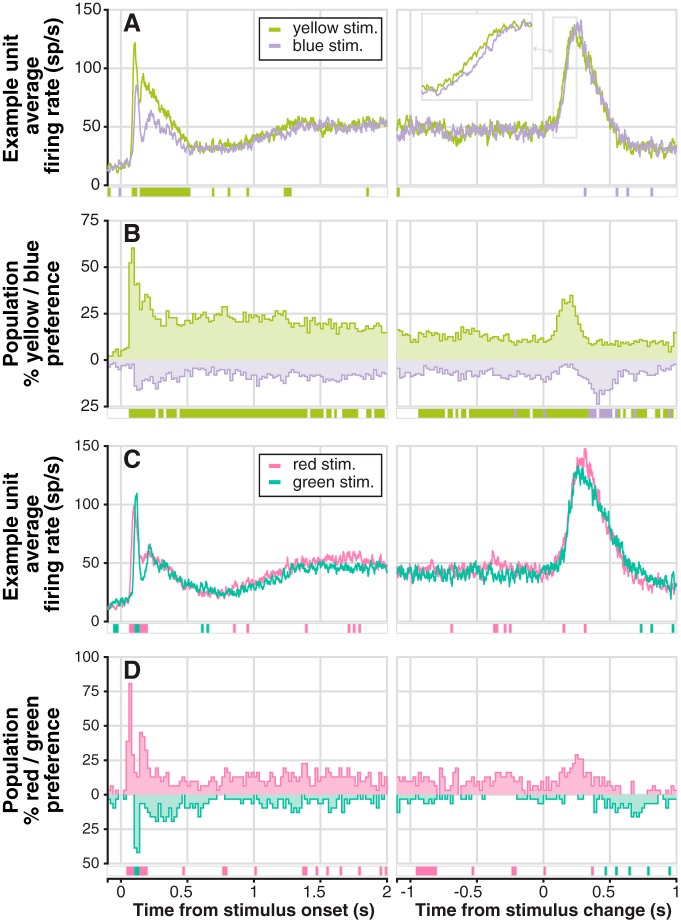
Example unit and population-level color preferences between color-pairs. ***A***, Example unit average firing rate traces aligned on stimulus onset (left) and color change (right), for yellow and blue stimuli. Significant preferences for one of the two colors in each bin is indicated by the row of colored boxes below; significance was determined using bootstrapped ROC areas comparing spike counts from nonoverlapping 20 ms bins (see Materials and Methods). Right panel inset shows a time-expanded view of the outlined rising portion of change-related phasic activity. ***B***, Population-level percentage yellow/blue preference (*n* = 139): the percentage of individual units with a significant preference in each bin; the row of colored boxes below mark bins in which the proportion of units preferring one of the colors was significantly greater than the proportion preferring the other as measured by χ^2^-proportion tests. ***C***, Example unit average firing rate traces for red and green stimuli (conventions as in ***A***). ***D***, Population-level percentage red/green preference (*n* = 31): conventions as in ***B***.

**Figure 3.**
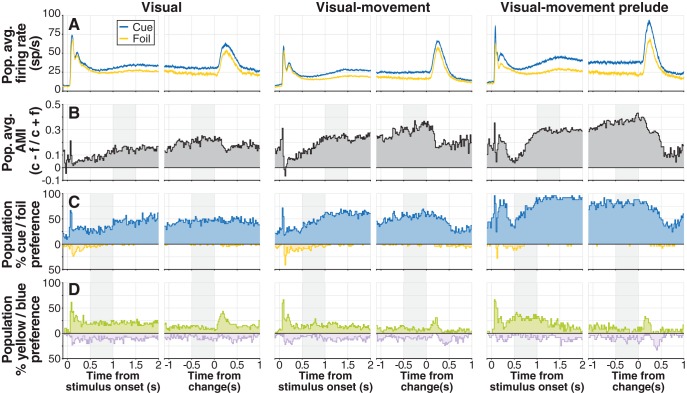
The strength of cue-related modulation varies by unit-type category in distinct ways from color-related modulation. ***A***, Population average firing rates for cued stimulus (blue) and foil (yellow) aligned on stimulus onset (left) and change (right), for V (*n* = 42; far left), VM (*n* = 45; middle), and VMp (*n* = 46; far right) categories. ***B***, Population average AMI by category: for each unit, average spike count in nonoverlapping 20 ms bins for the cued stimulus (c) and the foil stimulus (f) was used to calculate AMI = (c − f)/(c + f); individual AMIs were then averaged to obtain population AMI. Gray shaded regions mark time windows used for ANOVA (see Results). ***C***, Population-level percentage cue/foil preference by category: the percentage of units in each bin with significantly larger spike counts for the cue (blue) or the foil (yellow); significance was determined with bootstrapped ROC area. Shaded regions indicate time windows used for ANCOVA. ***D***, Population-level percentage yellow/blue preference by category: the percentage of units in each bin with significantly larger spike counts for the yellow or the blue stimulus determined with bootstrapped ROC area. Because of the similarity of individual neuron responses to increases and decreases, data from increase trials and decrease trials were pooled.

Because we used a limited number of hues (Fig. 1*D*), more traditional tuning curves (e.g., White et al., 2009, their Fig. 1) would have been difficult to interpret. Instead, we characterized individual neuron color preferences by comparing evoked activity between the color pairs “yellow”/“blue,” and “red”/“green” (Fig. 1*E*), which lie along two of the axes of the DKL colorspace (Derrington et al., 1984). A neuron’s preference in each pair was computed from spike counts in nonoverlapping 20 ms bins (aligned either on stimulus onset or change). For each unit, receiver operating characteristic (ROC) area was calculated between spike counts in each bin for yellow stimulus presentations and counts for blue stimulus presentations (Fig. 1*E*). A bootstrapping procedure was used to compute the confidence interval on ROC area in each bin, and if that confidence interval was completely above or below 0.5, the preference in that bin was considered significant; the position (above or below) dictated whether the preference was for one or the other member of a pair. The confidence interval was the values marking the 2.5th and the 97.5th percentiles of a 10,000 sample bootstrapped distribution of ROC area. Each sample of the distribution was an ROC area computed on spike counts drawn with replacement from the originals without combining them. Population preferences were the proportion of units with a significant preference for a color in each bin (Fig. 2*B*,*D*). A comparable method, bin-by-bin estimation of bootstrapped ROC area confidence interval, was used to compare activity for the cued stimulus to that evoked by the foil (Fig. 3*C*), activity evoked by saturation increases to decreases (Fig. 5*B*), and of hits to that of misses (Fig. 6*B*). These analyses follow common practice in the analysis of neuronal data by choosing time-windows of interest, dividing each window into bins, and interrogating each bin for statistical significance. Because the number of tests is not determined by the structure of the data itself but arbitrarily by the choice of time window and bin duration, we chose not to correct these tests. However, it is important to point out that the risk of Type I errors (false positives) is increased by conducting multiple tests.

A χ^2^-proportion test was used to compare population preferences for one or the other member of a color pair, as well as to compare cue/foil activity and hits/misses activity at the population level. This test evaluates the hypothesis that two or more proportions are different with the null hypothesis that the proportions are the same (Fleiss et al., 2013).

An attention-related modulation index (AMI) was computed for individual units from spike counts in 20 ms nonoverlapping bins for cue and foil stimuli as: cue – foil/cue + foil. Individual AMIs were averaged to obtain the population AMI (Fig. 3*B*).

To compare peak onset-evoked and change-evoked firing rates across neurons, we extracted each neuron’s maximum firing rate 50–200 ms after stimulus onset and 100–300 ms after stimulus change from average firing rate traces; different time windows were used to account for the slower time course of change-evoked compared with onset-evoked activity.

To compare change-detection activity for hits to change-detection activity for misses, we chose a time window comprising the top 90% of the peak of change-detection activity. We first found the maximum of the change-aligned firing rate, averaged across all 139 units. Then, we located the first point before and the first point after the peak where the firing rate trace crossed 90% of the maximum, these being 190 and 290 ms. This window was also used to assess the significance of change-detection activity in individual units: we compared the spike counts in the peak window to the spike counts in a baseline window extending from −100 to 0 ms relative to the change using a Wilcoxon rank sum test.

To quantify the contribution of several factors to differences in the timing of change-detection activity observed in different reaction time quantiles, we used a “hinge” model that has been used previously to determine the onset latency of smooth pursuit eye movements (Krauzlis and Miles, 1996; Adler et al., 2002). This model had three parameters: baseline offset, “start-of-rise” time, and “rate-of-rise”; baseline offset was fixed to the average firing rate in the 100 ms immediately preceding the color change, while start-of-rise time and rate of rise were fit to the rising portion of the change detection activity. We selected the rising portion of the change-detection activity using the mean baseline firing rate (μ_b_), the peak firing rate (p), and the standard deviation of the baseline firing rate (σ_b_), by finding the portion of activity satisfying: μ_b_ + 2σ_b_ < activity < p − σ_b_. The rate-of-rise was the slope of a line fit to the selected rising portion of the change-detection activity with linear regression, and the start-of-rise time was the time where the fitted line had a value equal to the baseline mean (Fig. 7*B*).

To determine the proportion of joystick release time (jRT) accounted for by the timing of change-detection activity in each unit, we followed a method based on Thompson et al. (1996). We split the distribution of jRT for hits into three quantiles (fast, 0–33rd percentile; medium, 33rd–66th percentile; and slow, 66th–100th percentile; Fig. 7*A*), and fit the hinge model to the average firing rate of each (Fig. 7*B*). Change detection activity timing was quantified by setting a threshold firing rate to the smallest “end-of-rise” (p − σ_b_) over the three quantiles, and finding the “threshold time” when each quantile’s hinge-fit crossed threshold. For each unit, we calculated the proportion of jRT accounted for by threshold time (Fig. 7*F*) as the average slope of the relationship between threshold time and median jRT (Fig. 7*E*); average slope was determined from the slopes of the two lines connecting the three data points in Figure 7*E*, producing a single value for each neuron in Figure 7*F*. The proportion of jRT accounted for by start-of-rise, rate-of-rise, and baseline offset values were calculated similarly: by taking the average slope of the relationship between the value and median jRT (Fig. 7*G–I*).

## Results

Two monkeys performed a covert spatial attention task requiring them to release a joystick in response to saturation changes at a cued location and to maintain their hold for changes at an opposing foil location or if no changes occurred (Fig. 1*A*). Hit rates (79% and 81%) and false alarm rates (6% in both) were comparable in the two animals (Fig. 1*B*). In 136 sessions, we recorded extracellular activity from 139 neurons throughout the SC. These neurons were found between 0.25 and 3 mm from the surface of the SC, primarily in the intermediate and deep layers, and the majority (*n* = 113) were categorized as V (*n* = 42), VM (*n* = 45), or VMp (*n* = 26), as defined in Materials and Methods. RF or movement field centers were at 6–14° of eccentricity, and ±60° from the horizontal.

During the task, SC neuron activity had several components: (1) phasic responses to task events, (2) modulation by cue condition, (3) modulation by stimulus color, and (4) information about response choice and response timing. We start by characterizing how SC activity varied with stimulus color.

### Color preferences of SC neurons during the task

The activity of SC neurons during the attention task was somewhat different for differently colored stimuli, and the magnitude of these color preferences varied throughout the trial. The example neuron in Figure 2*A* had higher firing rate for yellow than for blue directly after stimulus onset and persisting until 500 ms after (Fig. 2*A*, left). Both yellow and blue stimulus change events evoked large phasic increases in firing rate (Fig. 2*A*, right), with the activity for yellow rising slightly earlier than for blue (Fig. 2*A*, right, inset). The same example neuron responded earlier for red than for green at stimulus onset, but the peak onset response was larger for green (Fig. 2*C*, left). This neuron also showed slightly larger peak activity for red saturation changes than for green (Fig. 2*C*, right). We quantified individual unit color preferences using bootstrapped ROC area (see Materials and Methods), and combined individual preferences to determine population-level preferences.

Population-level color preferences were strongest at stimulus onset. Approximately 60% (84/139) of neurons showed a preference for yellow over blue at stimulus onset (80–100 ms bin; Fig. 2*B*, left), and 80% (25/31) preferred red over green (60–80 ms bin; Fig. 2*C*, left). Preference for yellow over blue settled at just under 25% of neurons ∼250 ms after onset (Fig. 2*B*, left), and later decreased slightly until just after the stimulus change event (Fig. 2*B*, right). In contrast, a variable and lower percentage of neurons preferred red over green from 250 ms after onset (Fig. 2*D*, left) up to the change event (Fig. 2*D*, right). The increased preference for yellow following stimulus change (Fig. 2*B*, right) stems from a tendency for yellow change-evoked activity to rise earlier than blue (as in Figure 2*B*, right, inset). A similar difference in timing also explains the small increase in red preference over green just after the change (Fig. 2*D*, right). To quantify overall population preferences, we compared the proportion of units exhibiting a preference for yellow versus blue and red versus green in each bin with a χ^2^-proportion test. The preference for yellow over blue was significant in 153/210 bins, with significant preference for blue over yellow in just 13/210 (all significant χ^2^ > 3.95). Meanwhile, the preference for red over green was significant in 31/210 bins, and for green over red in 7/210 (all significant χ^2^ > 3.96; see Materials and Methods for a note regarding Type I errors and large numbers of bins).

Overall, SC neurons showed modest color preferences that were strongest at stimulus onset and around the time of the stimulus change. We next describe the much stronger modulatory effects of the spatial cue, and briefly contrast the effects of cue and color.

### Cue-related modulation

Cue-related modulation was evident for neurons in each of the three main categories of SC neurons we studied, and this cue-related modulation was largest for VMp neurons (Fig. 3*A*).

We measured cue-related modulation in two ways: (1) we computed an attention modulation index (AMI = cue – foil/cue + foil), and (2) we computed the percentage of units in each category showing a significant preference for the cue over the foil using bootstrapped ROC area. Notably, AMI increased in the 500 ms before the window of time during which color changes were possible (1–4 s after stimulus onset), then appeared to ramp up slowly, reaching a maximum just before the stimulus change. On average, AMI was smallest for V, larger for VM, and largest for VMp neurons (Fig. 3*B*). Similarly, the percentage of units with a significant preference for the cue was smallest for V, larger for VM, and largest for VMp neurons (Fig. 3*C*).

We quantified the differences in AMI across unit types with two ANOVAs, one in a window aligned on stimulus onset (1–1.5 s) and one on change (-0.5 to 0 s). We found a significant effect of category on AMI in both ANOVAs (onset window: *F*_(2110)_ = 5.95, *p* < 0.01; change window: *F*_(2110)_ = 4.49, *p* < 0.05). *Post hoc* testing demonstrated that AMI for both VM and VMp was significantly greater than V, but not different between VM and VMp (α = 0.05). We also assessed the significance of any differences in population percentage cue preference across neuron categories with a χ^2^-proportion test in each of the 105 20 ms time bins covering the 2.1 s shown in Figure 3*C*. In a small number of bins, a larger percentage of VM neurons had a significant cue preference than V neurons (16/105 onset aligned bins, 3/105 change aligned; all significant χ^2^ > 3.92), but in contrast to the ANOVA results, VMp neurons had a greater percentage cue preference than VM in a large number of bins (73/105 onset aligned, 48/105 change aligned; all significant χ^2^ > 3.84). Thus, despite showing a statistically similar magnitude of cue-related modulation, VMp neurons were more likely to be significantly modulated by the cue than VM.

Cue preferences and color preferences changed in distinct ways during the trial. To compare the time course of cue and color preferences, we computed category-specific yellow-blue preferences across neurons (Fig. 3*D*). Both cue preference and color preference (yellow over blue) showed an onset transient at stimulus onset, but differences emerged later in the trial. In the window from 0.5 to 1 s after onset, cue preference increased while color preference was static. In the window from −0.5 to 0 s before the stimulus change, cue preference was static or increased while color preference decreased. Immediately following the stimulus change, there was a transient increase in color preference while cue preference decreased. We quantified the opposing trends in preference during the 0.5 to 1 s after onset window and −0.5 to 0 s before change window using ANCOVA. Both included factors of preference type (cue or yellow), unit type (V, VM, or VMp), and interaction terms. In both ANCOVAs, the interaction between time and preference type was significant (onset window: *F*_(1140)_ = 74.111, *p* ≪ 0.01; change window: *F*_(1140)_ = 15.092, *p* < 0.01), supporting the interpretation that cue and color preferences did not change in unison during the task.

### Change-detection activity

The foregoing analysis examined color preferences and cue-related modulation of onset-aligned and change-aligned SC activity. We now focus on the phasic increase in firing rate evoked by the saturation change in the color stimulus, which we term “change-detection activity”; this activity was significant in the vast majority of neurons (96%, 133/139; Wilcoxon rank sum test, all *p* < 0.05)

**Figure 4.**
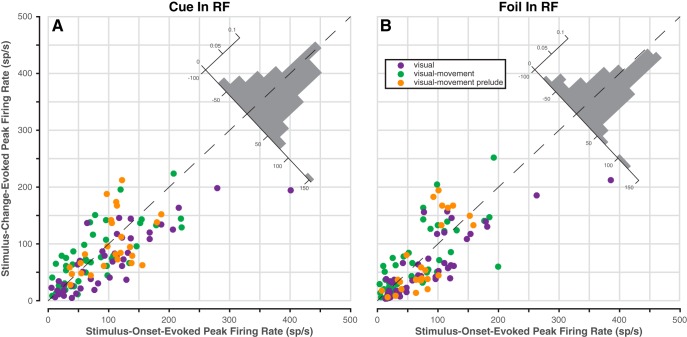
Peak change-evoked activity is comparable to onset-evoked activity. Data from V, VM, and VMp neurons only (*n* = 113). ***A***, Change versus onset peak activity when the cued stimulus was in the RF. Onset peak is the maximum average firing rate during the 50–200 ms after stimulus onset; change peak is the maximum during the 100–300 ms after stimulus change. Inset histogram is the distribution of distances from the dashed identity line. Data from increase trials and decrease trials were pooled.

Despite the fact that the saturation change was adjusted to be near each monkey’s detection threshold, the magnitude of change-detection activity was about the same as stimulus-onset activity. Irrespective of unit-type category (V, VM, or VMp), the peak of the average firing rate evoked by the stimulus change was similar to the peak evoked by stimulus onset both for the cued stimulus (Fig. 4*A*) and for the foil (Fig. 4*B*). To determine quantitatively whether there was any systematic variation in onset-evoked or change-evoked peak firing rate that depended on neuron category, stimulus identity, or combinations thereof, we conducted a three-way ANOVA on single neuron peak firing rates (see Materials and Methods). The factors in this ANOVA were (1) trial epoch (onset or change), (2) stimulus identity (cue or foil), and (3) unit-type category (V, VM, or VMp). The effect of trial epoch was not significant (*F*_(1140)_ = 2.36, *p* = 0.13), nor were any of the interaction terms (all *p* > 0.08), demonstrating that across our population of neurons there was no difference between onset-evoked and change-evoked peak firing rate, despite the fact that the stimulus saturation changes were small (0.03–0.05 DKL units) and gradual (80 ms duration), unlike the large luminance contrast and abrupt onset of the color patches.

**Figure 5.**
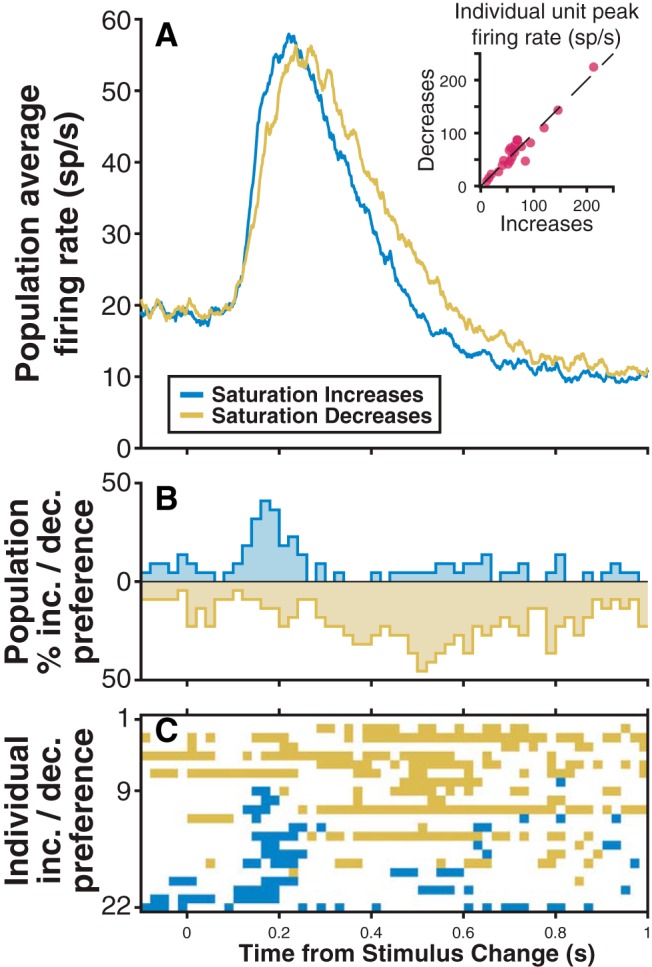
Change-detection activity is comparable for saturation increases and decreases. ***A***, Population (*n* = 22) average firing rates for saturation increases (blue) and decreases (gold). Inset is a scatter plot of maximum change-detection activity (in the 100–300 ms after change). ***B***, Population percentage preference for increases/decreases: the percentage of units with significantly larger spike counts for increases or decreases in each 20 ms bin; significant individual unit preferences were determined by bootstrapped ROC area. ***C***, Individual unit preferences for increases or decreases sorted by the total number of bins with significantly greater spike counts for saturation increases.

Change-detection activity was similar for saturation increases and saturation decreases. For a small set of isolated neurons (*n* = 22), we alternated blocks of saturation-increase trials and saturation-decrease trials, choosing stimulus parameters to maintain comparable hit and false-alarm rates for the two types of change. The magnitude of change-detection activity was comparable between saturation increases and decreases in the population average firing rate (Fig. 5*A*), and also for individual neurons (Fig. 5*A*, inset). A paired *t* test revealed that peak change-detection activity for saturation increases was not different from the peak for saturation decreases (*p* = 0.89). However, the average response to saturation decreases appeared to have slower temporal dynamics than saturation increases. Accordingly, across the population of neurons there was a preference for saturation increases between 100 and 200 ms after the change, followed by a preference for saturation decreases from ∼300 to 900 ms (Fig. 5*B*). The pattern of preferences for individual neurons suggests that these effects were largely separable: one subset of units showed a transient early preference for saturation increases (neurons 9–22), and the remainder showed an overall preference for saturation decreases (neurons 2–8; Fig. 5*C*).

### Behavioral information in change-detection activity

**Figure 6.**
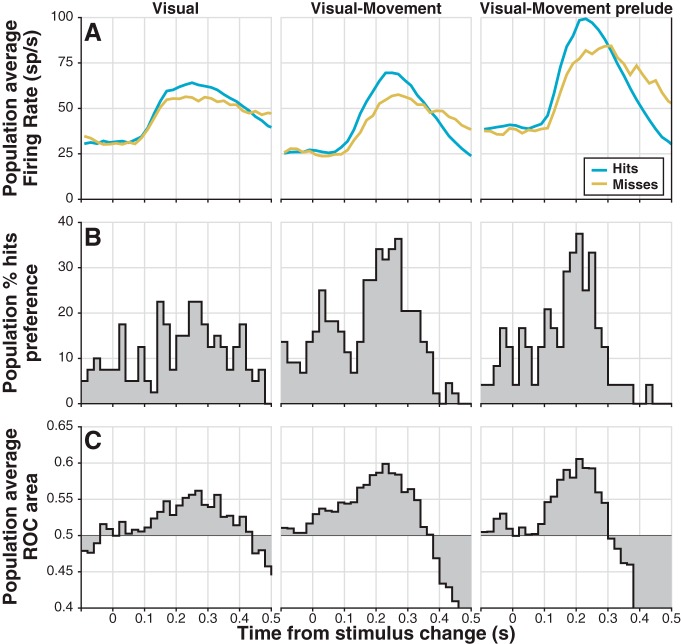
Change-detection activity is predictive of behavioral choice. ***A***, Population average firing rate traces for hits (blue) and misses (gold). After averaging spike trains convolved with a waveform resembling a postsynaptic potential (see Materials and Methods), activity in nonoverlapping 20 ms bins was averaged for comparison with preferences in ***B*** and ROC area in ***C***. ***B***, Population percentage hits preference: the percentage of units in each bin with significantly larger spike counts in hit trials versus miss trials; significance was determined by bootstrapped ROC area. ***C***, Population average ROC area or detect probability: average ROC area from the bin-by-bin comparison of spike counts for hits to those for misses. Data from increase trials and decrease trials were pooled.

Hits were associated with larger change-detection activity than misses. For all categories of units (V, VM, VMp), average change-detection firing rate was higher when it preceded a hit (i.e., correct joystick release) than when it preceded a miss (Fig. 6*A*). The difference in average firing rate for hits and misses emerged at the onset of change-detection activity and was maximal at the peak evoked firing rate. We quantified these differences between hits and misses in two ways: (1) we calculated the percentage of neurons in each category with significantly greater activity for hits than misses (from bootstrapped ROC area), and (2) we calculated the population average ROC area, referred to as “detect probability” (Cook and Maunsell, 2002; Nienborg and Cumming, 2009). VM and VMp neurons were both more likely than V to show significantly increased activity for hits over misses (Fig. 6*B*), and showed a larger detect probability (Fig. 6*C*). To quantitatively compare activity for hits and misses, we performed a two-way ANOVA on spike counts in a time window comprising the top 90% of the peak of change-detection activity (190–290 ms). The factors in the ANOVA were: (1) behavioral response (hit or miss), and (2) unit-type category (V, VM, or VMp). Both factors proved significant (behavioral response: *F*_(2220)_ = 7.38, *p* ≪ 0.01; unit-type: *F*_(1220)_ = 3.93, *p* < 0.05), but their interaction was not (*F*_(2220)_ = 0.26, *p* = 0.77). The lack of significance of the interaction term suggests that the peak difference between hits and misses did not differ significantly among the categories of units. We also compared the proportion of neurons with a significant preference for hits versus misses across categories with a χ^2^-proportion test; this test was run in each of several 20 ms bins (Fig. 6*B*), and also in the 190–290 ms ANOVA window. Although VM and VMp neurons showed larger percentages of neurons with a significant preference (Fig. 6*B*), in agreement with ANOVA results, % hits preference did not differ significantly across categories (bins: all χ^2^ < 5.26, all p > 0.07; ANOVA window: χ^2^ = 2.78, *p* = 0.25).

**Figure 7.**
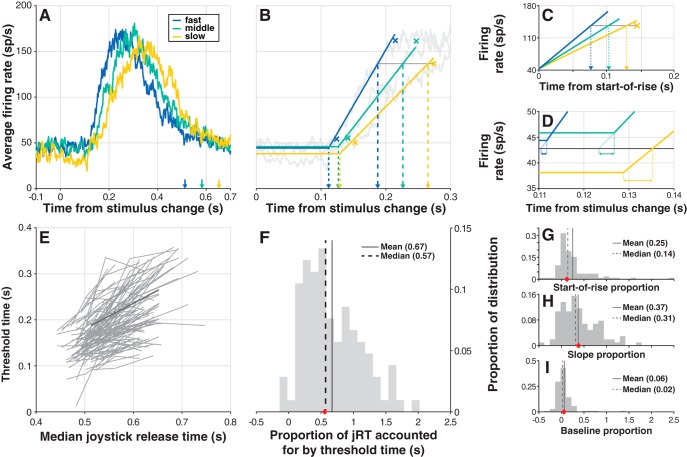
Timing of change-detection activity predicts jRT. ***A***, Example unit average firing rate for hits falling into each of three jRT quantiles: slow (yellow), medium (green), and fast (blue). Colored arrows indicate the median in-quantile jRT. ***B***, Example hinge model fits to the data in ***A*** (solid colored lines; see Materials and Methods). Colored Xs near hinge points indicate +2 SD from baseline, while those near the plateau of activity indicate −1 SD from the peak; the yellow X and connected gray line are the criterion firing rate value (minimum of the three −1 SD points) used to calculate threshold times, which are indicated by larger dashed lines. ***C***, To selectively determine the effect of differences in hinge model slope on threshold time, hinge models were aligned at hinge points and the “slope time” time at which each crossed the criterion firing was found (dashed lines). ***D***, To determine the effect of differences in baseline firing rate on threshold time, the “baseline time” required to go from each quantile’s baseline offset to an across-quantiles baseline mean was determined (horizontal double-arrowhead lines). ***E***, The relationship between threshold time and median jRT for each unit; this analysis follows the method of Thompson et al. (1996). Data from the example unit shown in ***A*** is plotted in darker gray. ***F***, Histogram of the proportion of jRT accounted for by threshold time. Each unit’s proportion is defined as the average slope of the two line segments belonging to that unit in ***E***, so that each neuron contributes one value to the distribution in ***F***; the red diamond on the abscissa is the proportion for the example unit in ***A***. ***G–I***, Proportion of threshold time accounted for by start-of-rise time (***G***), slope time (***H***), and baseline time (***I***): each unit’s proportion is the average slope of the relationship between the quoted time variable and median jRT; the red diamond is the example unit’s value. Data were pooled across increase trials and decrease trials.

The timing of change-evoked activity accounted for a substantial proportion of jRT. To illustrate this, we divided the data from hit trials in each session into three latency quantiles (fast, middle, and slow), and plotted average firing rates for each (Fig. 7*A*). The clear difference in the timing of firing rate activity along with joystick release time (jRT) led us to ask two questions. (1) What proportion of jRT could be accounted for by the timing of firing rate activity? And (2) what factor or factors gave rise to differences in the timing of the firing rate activity? To address these questions quantitatively, we fit a simple hinge model to each unit’s average firing rate data for each quantile (Fig. 7*B*). This model consisted of a constant baseline portion and a linear-rise portion (see Materials and Methods). The constant baseline was set to the average firing rate in the 100 ms preceding the stimulus change. The linear-rise portion was the least-squares fit to the segment of the average firing rate trace between 2 SD above baseline and 1 SD below the peak (SD calculated from the 100 ms before change). We termed the intersection point of the baseline and linear-rise portions “start-of-rise”, and the −1 SD from peak values “end-of-rise”. The smallest end-of-rise value for each unit was used as a criterion firing rate value, and the “threshold time” at which each quantile’s hinge-fit crossed this value was calculated.

Using this model, we found a clear trend in the relationship between the threshold time and the median in-quantile jRT for each unit (Fig. 7*E*). On average, threshold time accounted for 67% of the jRT (Fig. 7*F*): if the median jRT of the fast and slow groups differed by 100 ms, their threshold times would differ by 67 ms. We next considered the three factors that contribute to threshold time differences in the hinge model; these factors are: (1) start-of-rise (Fig. 7*B*), (2) rate-of-rise (Fig. 7*C*), and (3) baseline offset (Fig. 7*D*). We computed the relative contributions of these components (Fig. 7*G–I*) and found that rate-of-rise accounted for a substantially greater proportion of the total than the other two factors. A one-way ANOVA on these proportions revealed a significant main effect (*F*_(2312)_ = 21.38, *p* ≪ 0.01), and that differences in rate-of-rise among quantiles contributed a significantly greater proportion than did baseline differences or start-of-rise differences (Tukey-Kramer *post hoc* tests, α = 0.05). Thus, the timing of change-detection activity, in particular, the rate-of-rise after the saturation change, accounted for more than half of the jRT, even though the joystick was released more than 200 ms after this phasic activity.

## Discussion

We have shown that neurons in the primate SC exhibit large phasic increases in firing rate shortly after near-threshold changes in color saturation. This change-detection activity was as large as the stimulus-onset response, and was evoked by both saturation increases and decreases. Change-detection activity was significantly larger when monkeys released the joystick (hits) than when they maintained their hold (misses), and its timing accounted for reaction time despite occurring several hundred milliseconds before joystick release. The same neuron types whose change-detection activity best predicted behavioral choice (VM and VMp) also showed the largest cue-related modulation throughout the trial. We also found modest color preferences along the cardinal axes of DKL color space (“blue-yellow” and “red-green”), with the population preferring yellow over blue and red over green.

### Change-detection activity integrates event detection and behavioral relevance

Change-detection in our color task is a novel example of the way SC neurons indicate the time and location of behaviorally relevant visual events. The phrase “behavioral relevance” unifies diverse modulatory effects on SC activity, for example: spatial cueing (Ignashchenkova et al., 2004), target probability (Basso and Wurtz, 1998), and target identity (McPeek and Keller, 2002). These effects have primarily been seen as supporting orienting or targeting behaviors. We instead speculate that SC activity guides action selection more generally by signaling which sensory data should be used to choose an appropriate response to a visual event.

Supporting this interpretation, we found SC activity was not only modulated by behavioral relevance but was also predictive of the joystick release, arguably neither an orienting nor a targeting response. Change-detection activity was larger for the cue than for the foil stimulus, but cued change-detection activity was larger still for hits than for misses (Fig. 6). We also found that the timing of change-detection activity accounted for a substantial portion (67%) of the joystick release latency. These findings demonstrate that SC activity indicates if and when the monkey will release the joystick in response to a stimulus change.

Previous work in SC has characterized its role as reporting novel visual onsets primarily at the service of orienting. Lettvin et al. (1961) roughly partitioned SC neurons in the frog as responding either to “newness” or “sameness” based on habituation to stimulus persistence or repetition. Boehnke et al. (2011) described a subpopulation of macaque SC neurons as detecting novel events, finding that both brighter and darker oddballs evoked responses with stronger sustained components (∼75–90 to 200 ms) than nonoddballs. Boehnke and Munoz (2008) proposed that the visual onset response in macaque SC integrates stimulus information as well as relevance and expectation, and influences future actions and immediate orienting. Our results broaden this line of thought, showing that not only onsets, but also near-threshold changes can evoke SC activity, and that this activity can be used not only for orienting, but also for other nonorienting motor responses.

How might the SC accomplish this more general role? One possibility is that the SC reports the detection of salient events to the substantia nigra pars compacta (SNc). Dopaminergic neurons in macaque SNc respond to novel stimulus events or those associated with reward at short latency (<100 ms; Schultz, 1998). Rat SNc dopaminergic neurons have similar responses, mediated by the tectonigral projection from SC to SNc (Comoli et al., 2003; Dommett et al., 2005). Redgrave and Gurney (2006) hypothesize that salient event-detection signals from SC allow SNc to reinforce novel actions that caused the event: if an animal unintentionally triggers a rewarding sensory event, the behaviorally relevant event is reported to SNc by SC, and the preceding actions are reinforced by dopamine release in the striatum. To successfully perform our task, animals instead needed a reliable link between a behaviorally relevant event (cued saturation change) and a subsequent action (joystick release); the recruitment of SNc dopaminergic neurons by SC change-detection activity may have been important for learning that link between event and action.

Change-detection activity is unlikely to be a manifestation of previously observed reach-related activity in the SC and underlying mesencephalic reticular formation (Werner, 1993; Werner et al. 1997b; Stuphorn et al., 1999; 2000). The vast majority of our neurons had visual responses, which is uncommon in reach-sensitive neurons (Werner et al., 1997a). Also, reach-sensitive neurons are mostly found deeper than 3 mm from SC’s surface, but our neurons were all isolated between 0.25 and 3 mm. Lastly, reach-related activity generally persists during the movement and often after (Werner et al., 1997a; 1997b; Stuphorn et al., 1999; 2000), whereas change-detection activity terminated 100–200 ms before the joystick-release. Thus, our results are consistent with a role for the SC in processing visual events regardless of the effector used to respond (Nummela and Krauzlis, 2010).

### The relationship between change-detection and cue-related modulation

Among SC neurons, those with VM activity (i.e., VM and VMp) are most strongly implicated in the integration of cue-related and change-detection signals. These neurons showed both the largest cue-related modulation, consistent with previous studies (Kustov and Robinson 1996; Ignashchenkova et al., 2004) and best predicted joystick release. It is probable that cue-related enhancement of activity increases the likelihood of larger change-detection activity and thus of a joystick release, whereas cue-related reduction of foil activity leads to smaller change-detection activity and to joystick hold. Situated in the intermediate and deep layers of SC, these neurons could be the origin of event-detection signals sent to SNc (May et al., 2009), and are anatomically poised to integrate a variety of signals from visual and fronto-parietal areas of cortex (Lock et al., 2003). The excitatory inputs from cortex are also presumably the source of the color-change signals that were instrumental in our task.

The SC also receives inhibitory inputs from the substantia nigra pars reticulata (SNr), and we speculate that these may play an as-yet unappreciated role in modulating SC activity during attention tasks. The SNr projects selectively to intermediate and deep SC (Hikosaka and Wurtz, 1983; Jiang et al., 2003) where it modulates neuronal firing via GABAergic synapses (Isa et al., 1998; Kaneda et al., 2008). Inhibition from the SNr likely mediates reward-based modulation of saccade latency and peak velocity (Ikeda and Hikosaka, 2003), and phasic reductions in SNr neuronal activity have been observed for target-identifying luminance changes (Basso and Wurtz, 2002), but the SNr’s influence need not be limited to saccades. In our task, similar SNr inhibition could be responsible for reducing the excitability of SC neurons responding to the foil stimulus, so that they emit lower activity for behaviorally irrelevant events.

### The SC and color

We used color in our task because it offers a good test of whether the SC’s role in the control of spatial attention extends across visual features. Earlier work in anesthetized monkeys concluded the retinotectal projection was not color opponent (Schiller and Malpeli, 1977; de Monasterio, 1978), the indirect geniculostriate input to SC was not color opponent (Schiller et al., 1979), and that SC neurons are chromatically nonopponent (Marrocco and Li, 1977). More recently, work in awake behaving monkeys, perhaps a key difference, found evidence consistent with cortically derived color-opponent inputs to SC (White et al., 2009) and S-cone inputs (Hall and Colby, 2014). White et al. (2009) found that SC neurons respond to isoluminant color stimuli across a range of hues, and speculated that SC’s color information might come from cortical area V4, or from other geniculostriate input. Challenging the widespread belief that S-cone stimuli are “invisible” to the SC (Sumner et al., 2002), Hall and Colby (2014) found that “nearly all visual SC neurons” respond to S-cone isolating stimuli, and suggested that SC’s S-cone sensitivity might arise from previously underappreciated components of the retinotectal projection.

Saturation-change information in the SC may derive from neurons in the “globs” of macaque posterior inferior temporal cortex and area V4. Glob neurons have several properties that argue for their importance in color perception (Conway et al., 2007; Conway and Tsao, 2009; Namima et al., 2014), and modeling work has concluded that their responses are consistent with a sensitivity to saturation (Bohon et al., 2016). V4 neurons are known to show vigorous phasic activity shortly after isoluminant changes in color (Womelsdorf et al., 2006; Fries et al., 2008, Zhou et al., 2016), and to respond strongly to orientation changes (Cohen and Maunsell, 2010). The activity evoked by orientation changes is suggestive of a generic change-detection signal, because it occurs even when the changed orientation is not at the peak of the neuron’s tuning for orientation. These V4 signals could influence SC activity by a direction projection to the intermediate and deep layers (Lock et al., 2003), but whether glob neurons project to SC is unknown.

In summary, visual cortical areas are likely required to generate the saturation-change activity we found in SC neurons, but the mechanisms that give rise to this phasic activity in the SC have not yet been identified. Saturation-change activity in SC is unlikely to result from intrinsic processing for several reasons. Saturation-change activity was evident in neurons throughout the layers of SC, which receive very different inputs: retinotectal projections exclusively target superficial layers (Schiller and Malpeli, 1977), and distinct cortical areas target superficial compared with intermediate and deep layers (Lock et al., 2003). Saturation-change activity was present in neurons with different functional properties: purely V, VM, and VMp neurons all showed this response. Individual neurons showed saturation-change activity for saturation increases and decreases and along multiple directions in DKL color space. Instead, the color-change activity we found with our stimuli appears to involve a mechanism that reads out visual cortical activity specifically for the purpose of detecting changes, and provides signals to the SC consistent with its long evolutionary history of detecting behaviorally relevant events.

### Conclusion

We have shown that subtle changes in color saturation evoke vigorous phasic increases in firing rate in primate SC neurons in the absence of orienting behaviors. Change-detection activity is both modulated by spatial cueing, and predictive of manual choice behaviors. We conclude that change-detection activity in SC is suitable to guide action selection, regardless of the visual feature that is monitored or the specific motor response that follows.

## Synthesis

Reviewing Editor: Tatiana Pasternak, University of Rochester Decisions are customarily a result of the Reviewing Editor and the peer reviewers coming together and discussing their recommendations until a consensus is reached. When revisions are invited, a fact-based synthesis statement explaining their decision and outlining what is needed to prepare a revision will be listed below. The following reviewer(s) agreed to reveal their identity: Nate Hall.

This manuscript investigates the responses of superior colliculus neurons in a covert attention task that requires monkeys to report a change in the color saturation of a dynamic visual stimulus using a manual response, while ignoring changes in an

irrelevant "foil" stimulus. The stimulus is clever because SC neurons are normally fairly insensitive to variations in color, and it shows that a small change in color saturation leads to an increase in SC activity. They also show that this activity is behaviorally relevant since the increase is larger when monkeys correctly detected the change than when it was missed, and that the timing of the activity increase varied with the monkey's manual reaction time.

The revised manuscript addressed most of the reservations raised in the previous review. However, before it can be accepted for publication, the authors must address the issues raised by the attached review of the latest revision.

Issues to address:

1. The point about multiple comparisons

2. Clarify the latency analysis shown in Fig 7 as suggested by the reviewer

3. Comments concerning lines: 77-78; 181-184; line 379; lines 532-545

Reviewer 1

ADVANCES THE FIELD

The manuscript demonstrates that visual SC neurons are sensitive to

subtle changes in color saturation during a cued attention task

requiring a manual behavioral response. SC sensitivity is stronger for

cued stimuli and for correct trials. This advancement adds to the body

of evidence that the SC may participate in generalized attention/action

selection because color is a feature to which the SC is not particularly

selective and manual responses are not attributed to the activity of

visual and saccade responsive SC neurons. The present study further

provides support for previous hypotheses on the role of SC in generalized attention but uses a novel and more powerful approach.

STATISTICS

As explained in my comments, the analysis in Figure 7 is not clear

enough for me to answer this question.

I also feel obligated to point out the number of multiple comparisons

present in the paper. Reports of proportions of neurons with a

significant response and the bin-wise analyses have a substantial

number of multiple comparisons. These do not compromise the

integrity of the paper or its findings, but it is worth pointing out the

type 1 error rate in these situations.

COMMENTS TO THE AUTHORS

The manuscript is strong and improved overall. My only concern is my

understanding of the analysis presented in Figure 7. Otherwise I have

only a few small comments and suggestions.

The latency accounted for analysis presented in Figure 7 continues to

be either confusing or misleading. Throughout the manuscript,

including the abstract, statements about variation in jRT and the

amount of variation accounted for by SC activity are made. If I

understand the analysis correctly, these statements should refer to the

proportion of jRT attributable to neuron response latency in the SC (as

the axes in Figure 7F-I are labeled). This analysis shows that about

67% of the total behavioral reaction time, jRT, takes place at or before

the level of SC neuronal threshold crossing. It does not say anything

about the variation in jRT accounted for by SC response latency.

Accounting for the variance in jRT responses would require a Pearson

correlation on the data shown in Figure 7E and the corresponding r2

value. The analysis as presented instead computed the regression

slope of the data in Figure 7E which describes the expected increase in

jRT given an increase in SC response latency. The variance in jRT

accounted for by variance in SC response latency for each neuron

would require a correlation analysis between the jRT and SC neuron

response latency calculated over each individual trial.

To reduce this confusion I would recommend removing all mentions of

variation from the manuscript. These should instead refer to the

proportion, or amount of jRT attributable to SC response latency.

Additionally, I still do not understand where the slope data come from.

Lines 359-363 and lines 553-554 suggest that hinge models were fit

separately to the average firing rate in each quantile for each neuron.

This would give 3 threshold times for each neuron and provide 3 data

points for each neuron in Figure 7E. The proportion of jRT accounted

for would then be the slope of the regression line fit to these 3 data

points. It would be questionable to fit a line to 3 data points. But lines

366-368 say that the proportion of jRT was computed as the average

slope of this relationship. The concept of average slope is repeated in

the Figure 7F legend. It is not clear what is being averaged here. The

data for the example neuron in Figure 7E (the dark gray line) does not

appear to match the threshold crossing times shown for this neuron in

panel 7B. The neuron in 7B clearly crosses threshold before 0.2 s

during the fast quantile, but its vertical axis value is clearly greater

than 0.2 s in panel 7E. This further convinces me that I have missed

an important point or that something is amiss.

Lines 77-78: Sentence says that Change-detection activity is

modulated by choice and latency but causality is presumably the

reverse.

Lines 181-184: I like the authors comment in the rebuttal "We

modified the task structure for the second monkey to correct an

inappropriate behavioral strategy that was not a problem with the first

monkey" more than what is here because it emphasizes a behavioral

control purpose but all data analysis trials were identical.

Line 329: I think the plus and minus operators are flipped in the AMI

formula.

Lines 331-334: It would be reassuring to state here that different time

windows were used (I am assuming) to account for the slower time

course of changes.

Line 379: It would be good to sate here that these were intermediate

layer neurons. I believe this has only been stated in the abstract to

this point.

Lines 532-545: This analysis made me want to know what percentage

of the SC units preferred hits vs. misses overall. Figure 6B shows that

about 35% of cells were significant at a given small time bin. But I

would assume that some cells are significant for some times bins and

not others, making the total percentage of SC neurons selective for

hits actually much higher than 35%? For example, if significance were

computed over a large bin such as that used for the accompanying

ANOVA what percentage of SC neurons would be selective for hits over

misses?
